# Total knee arthroplasty treatment of rheumatoid arthritis with severe versus moderate flexion contracture

**DOI:** 10.1186/1749-799X-8-41

**Published:** 2013-11-15

**Authors:** Denglu Yan, Jing Yang, Fuxing Pei

**Affiliations:** 1 Nanshan Hospital of Guangdong Medical College, Shenzhen 518052, China; 2 West China Hospital, Sichuan University, Chengdu 610065, China

**Keywords:** Total knee arthroplasty, Rheumatoid arthritis, Flexion contracture, Treatment

## Abstract

**Background:**

This study aims to explore the technique of soft tissue balance and joint tension maintenance in total knee arthroplasty (TKA) for the rheumatoid arthritis (RA) patients with flexion contracture of the knee.

**Methods:**

This retrospective study reviewed flexion contracture deformity of RA patients who underwent primary TKA and ligament and soft tissue balancing. Based on the flexion contracture deformity, the remaining 76 patients available for analysis were divided into two groups, i.e., severe flexion group (SF) and moderate flexion group (MF).

**Results:**

There were no intraoperative complications in this study. All patients had improved Knee Society Rating System scores and range of motion. The flexion contracture was completely corrected in MF and SF patients. There were no cases of patellar dislocation, but three cases had mild mediolateral instability in severe flexion group. Four knees (two knees in SF versus two knees in MF) had transient peroneal nerve palsy but recovered after conservative therapy.

**Conclusions:**

TKA can be performed successfully in the RA knees with severe flexion contracture. It is very important in TKA to maintain the joint stability in the condition of severe flexion contracture deformity of the RA knee.

## Introduction

Rheumatoid arthritis (RA) is a chronic inflammatory disorder characterized by synovial hyperplasia and resulting joint destruction. The knee is among the most commonly affected joints in RA, and it is estimated that up to 90% of patients with RA will eventually have the involvement of the knees [1]. Among those patients, progressive destruction of joints leads to the occurrence of flexion contracture in both of their knees and thus these patients are deprived of ambulation for long periods of time [2-4]. Underlying the severe flexion deformity is a usually complex combination of musculotendinous, ligamentous, and capsular contractures as well as often bone loss and significant valgus. Although the total knee arthroplasty (TKA) can be performed in this challenging patient [4,5], intraoperative correction of severe flexion deformity presented a challenging situation for orthopedic surgeons [4,6,7].

For RA patients, bone cuts have to be performed according to the anatomy and implant design and appropriate ligament balancing is required. However, it is potentially a poor strategy, as more bony cuts are needed to get the knee straight in the operation of RA patients with severe flexion contracture, which creates more problems with respect to instability thereby causing pain and dysfunction [8,9]. However, incomplete intraoperative correction of severe flexion deformity would lead to more residual flexion contracture postoperatively [10]. Therefore, proper soft tissue balancing was very important in TKA for RA patients with flexion contracture and valgus deformity, which do not only achieve an obvious correction of the flexion contracture but also effectively improve the range of motion and the functional recovery of the knee joint after TKA [11]. Atilla et al. [12] reported that pre-operation flexion contracture of 27.5° is an important threshold and patients should be operated before that stage to gain maximum benefit with minimal gait abnormalities. Mitsuyasu et al. [13] reported that flexion contracture eventually existed if the contracture was more than 15° 3 months after TKA surgery in severe flexion contracture of the knees. Cheng et al. [14] reported that patients with a preoperative fixed flexion deformity show continued improvement in their fixed flexion up to 10 years post-arthroplasty and have similar outcomes to those with no preoperative fixed flexion.

Along with our increased understanding of the RA patients with flexion contracture, special attention should be paid to the inferior bone density, and the soft tissue needs to be treated with special care. With the development of technique and device of TKA, it is recommended to limit bone resection with mandatory release of the posterior capsule and the collateral ligaments to get the knee straight in the operation and stable in the post-operation in the most severe cases [15]. Although it has been reported that the release of the posterior capsule and the collateral ligaments until some flexion for severe flexion contractures remains in RA patients, the debate continues as to which flexion contractures should be totally or partly corrected in operation [10,16-18]. It is very important for the modern TKA not only to restore a balance between the osteotomy and ligament release in procedures but also to maintain the joint tension in procedures to prevent joint laxity in the unusual condition of severe flexion contracture deformity of the RA knee. The purpose of this paper was to report our experience on RA knees with severe flexion contractures performed with one-stage TKA.

## Patients and methods

This retrospective study reviewed severe flexion contracture of RA patients who underwent primary TKA and soft tissue balancing from June 2006 to July 2008. The data included preoperative, intraoperative, and postoperative evaluation at standard intervals and annual follow-up reports. All patients were diagnosed as having RA according to the American College of Rheumatology criteria. The inclusion criteria were RA knees had flexion contracture with valgus deformity. Exclusion criteria included pathologic conditions of the RA knee (trauma, tumor, or infection). Eighty-four patients initially fulfilled the study criteria, and eight patients were lost to follow-up. According to the criterion of flexion contracture which is beyond or less 30°, the remaining 76 patients available for analysis were divided into two groups, i.e., severe flexion group (SF) (with flexion contracture beyond 30°; 36 cases initially, lost 2 cases, and 34 cases and 58 knees remain) and moderate flexion group (MF) (with flexion contracture less 30° and beyond 10°; 48 cases initially, lost 6 cases, and 42 cases and 64 knees remain). The data of age and sex distribution, flexion, range of motion (ROM) and knee society Rating System (KSS) score, and the course of disease are shown in Table 1.

**Table 1:** Patient data (data expressed as means ± SD)

**Group (case/knee)**	**Sex**		**Age (years)**	**Flexion (deg)**	**ROM (deg)**	**KSS**	**Course of disease (year)**
	**Male/knee**	**Female/knee**					
SF (34/58)	13/19	21/39	50.32 ± 8.69	50.84 ± 17.37	31.86 ± 11.25	27.48 ± 13.29	12.16 ± 2.25
MF (42/64)	14/20	28/44	48.68 ± 7.58	19.67 ± 10.46	68.16 ± 15.37	43.62 ± 15.46	9.30 ± 1.08

There was no significant difference between two groups (*p* > 0.05).

The TKA surgical procedure included a standard anteromedial approach, the use of an intramedullary femoral and extramedullary tibial alignment rod, measured bone resection, and differential ligament balance in flexion and extension [9]. Measured resection implies that the amount of bone resected from the intact compartment of the joint equals the thickness of the implant, while restoring correct alignment by resecting the bone perpendicular to the mechanical axis. Based on the correct osteotomy, recovering full extension at the end of surgery is mandatory, by first releasing the posterior capsule and the collateral ligaments from their osteophytes and secondly by extending the distal femoral cut where necessary. Once the correct bony alignment is achieved, the flexion and extension spaces are secured equally without massive soft tissue release and an additional distal femur cut. It is very important in procedures not only to restore a balance between the osteotomy and ligament release but also to maintain the joint tension to prevent joint laxity in severe flexion contracture of RA knees.

All patients received low molecular weight heparin as prophylaxis for deep vein thrombosis; the first dose was initiated 8 h after the operation. All patients received three doses of a second-generation cephalosporin and metronidazole as prophylaxis for infection, with the first dose administered at the induction of anesthesia. The same protocol for postoperative management was utilized in both groups, which included bedside continuous passive motion machine (CPM) therapy, physical therapy with partial weight bearing, and quadriceps and hamstring strengthening exercises starting on the second postoperative day. Splints are supportive devices for flexion in patients until the some residual flexion contractures were totally corrected.

**Figure 1:**
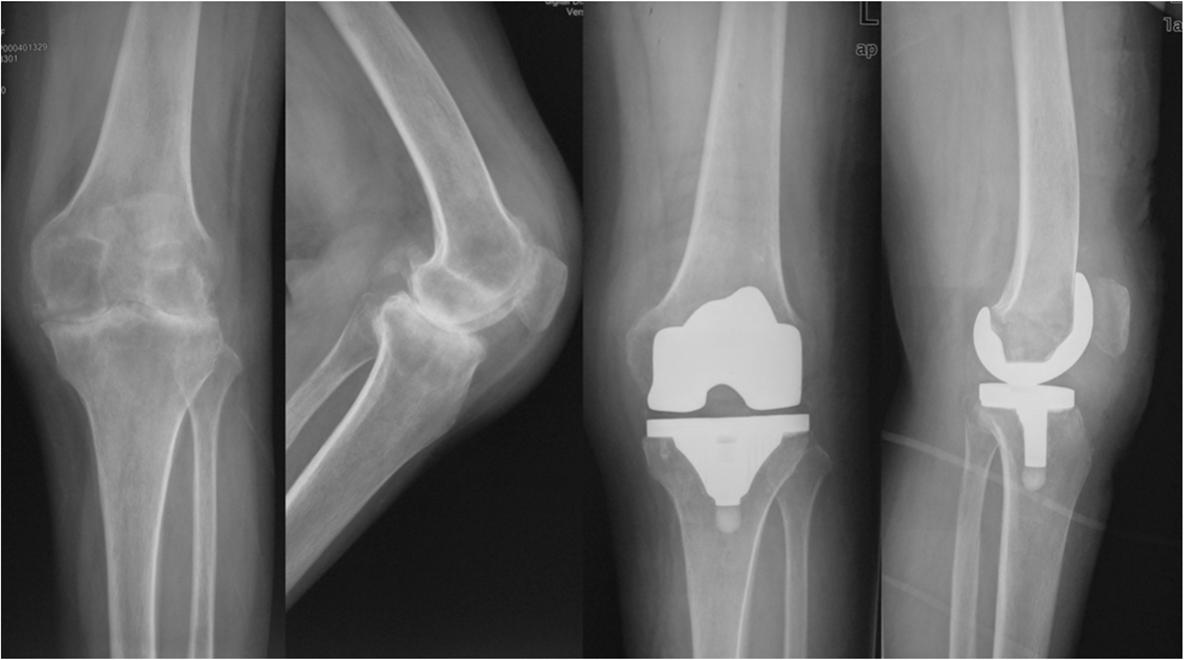
**Plain anteroposterior and lateral radiographs of a 43-year-old female which reveal joint destruction.** This case is a 43-year-old female patient with RA with flexion contractures deformity. After TKA on the right knee, the 27° flexion contracture was completely corrected postoperatively.

**Figure 2:**
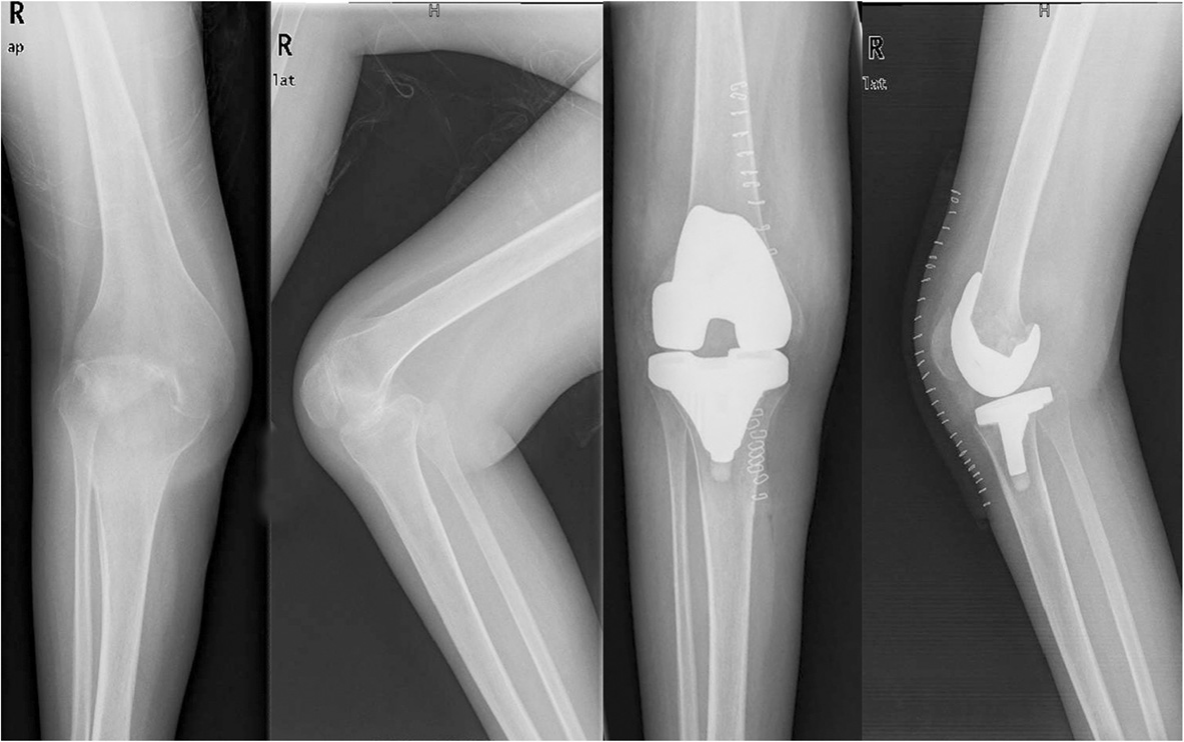
**1 Plain anteroposterior and lateral radiographs of a 49-year-old female which reveal joint destruction.** This case is a 49–year-old female patient with RA with flexion contractures deformity. After TKA on right knee, the 53° flexion contracture was completely corrected postoperatively.

The knees were assessed preoperatively and at yearly intervals after operation using KSS [19]. Furthermore, AP and lateral knee X-rays are performed to detect any radiolucencies using Canvas 9.01 software (Deneba Systems, Scientific Imagine Edition, Miami, FL, USA) to measure the deformity on the X-ray film (Figures 1 and 2).

### Statistics

All measurements were performed by a single observer and are expressed as means ± standard deviation (SD). Using the SPSS 16.0 statistical software; classic *t* test and Chi-square test were performed.

## Results

There were no intraoperative complications in this study. Soft tissue release surgery and additional bone cuts were performed in all cases of severe flexion contractures. Afterwards, 76 cases had follow-up (8 cases were lost) from 30 to 49 months (average of 39 months), with the average of 40.45 ± 5.26 months on SF and 39.88 ± 6.32 months on MF group (*p* > 0.05).

The flexion contractures and ROM were shown in Table 2. The average flexion contractures and ROM were not different between SF and MF groups (1.14 ± 0.27 vs. 1.12 ± 0.35 and 115.72 ± 15.13 vs. 118.34 ± 12.68). As shown in Table 2, the KSS improved and was better in MF group than SF group (87.15 ± 8.64 vs. 80.67 ± 9.35). Based on the Hospital for Special Surgery score, the rate of good or excellent was higher in MF group than SF group (SF = excellent, 27 knees; good, 20 knees; general, 8 knees; and poor, 3 knees; MF = excellent, 34 knees; good, 24 knees; and general, 6 knees).

**Table 2:** Clinical outcomes (data expressed as means ± SD)

**Group**	**Flexion (deg)**		**ROM (deg)**		**KSS score**	
	**Pre-OP**	**Post-OP**	**Pre-OP**	**Post-OP**	**Pre-OP**	**Post-OP**
SF	50.84 ± 17.37	1.14 ± 0.27^a^	31.86 ± 11.25	115.72 ± 15.13^a^	27.48 ± 13.29	80.67 ± 9.35^a^
MF	19.67 ± 10.46	1.12 ± 0.35^a^	68.16 ± 15.37	118.34 ± 12.68^a^	43.62 ± 15.46	87.15 ± 8.64^a^

^a^No significant difference in these groups (*p* > 0.05). OP, operation.

There were no infection complication and no cases with patellar dislocation or subluxation seen in this study. There were three cases with mild mediolateral instability in the SF for a massive release of soft tissue during TKA. Four knees (two knees in SF versus two knees in MF) had transient peroneal nerve palsy but recovered after conservative therapy. Five knees (two knees in SF vs. three knees in MF) suffered from complication of deep vein thrombus (DVT) within 1 week after operation and obtained good healing after active treatment. Figures 1 and 2 show plain anteroposterior and lateral radiographs.

## Discussion

RA knees with severe flexion contracture usually present with posterior subluxation of the tibia, proximal tibial bone deficiency combined with valgus deformity, and external rotation of the tibia, which can be partially attributed to the contracture and the traction of the biceps muscle and iliotibial tract [20]. The involvement of the periarticular soft tissues is part of the constellation of pathology in rheumatoid arthritis. Hence, it is critical to achieve correction of deformity, equalize the medial and lateral soft tissue tension, and implant the components accurately. Appropriate soft tissue balancing in the form of ligament and capsular release at the time of arthroplasty is essential to the success of the procedure [21,22]. As to the remaining some flexion in operation, it was especially important to properly position the individual components and the resulting overall alignment of the lower extremity in RA knee with one-stage TKA [18]. In the present study, successful TKA was performed in not only in moderate flexion contracture patients but also in severe flexion deformity of RA patients, and all cases had good clinical results. Once the correct bony alignment is achieved, it is very important for the success of TKA that the medial and lateral joint laxity does not exceed more than 2 mm in the stress test (varus and valgus stress testing) when prostheses are implanted.

Although TKA can be performed in this challenging patients [4,5], complete intraoperative correction of severe flexion deformity presented a challenging situation for orthopedic surgeons [4,6]. Various techniques of addressing these deformities have been described including additional bony resection, ligamentous releases, and the use of increasing constraint prosthesis [16]. However, an ideal soft tissue balance is difficult to obtain during surgery [23]. Appropriate soft tissue balancing in the form of ligament and capsular release at the time of arthroplasty is essential to the success of TKA procedures, which not only achieves an obvious correction of the flexion contracture but also effectively improves the range of motion and the functional recovery of the knee joint after TKA [9,20]. However, indications of orthopedic procedure on the flexion contracture were complex and required special consideration of the adequate collateral stability and extensive experience in TKA surgery [2,24-26]. In our early experience on severe flexion contractures in two RA patients, instability was caused by a massive release of soft tissue during TKA procedure. Therefore, appropriate soft tissue balancing in the form of ligament and capsular release at the time of arthroplasty is essential to the success of TKA procedures in severe flexion contractures of RA patients.

Flexion contracture is a common deformity encountered during total knee arthroplasty, and severe fixed deformities require surgical correction with release of the contracted soft tissues and appropriate management of the femoral bone resection [27]. Traditional methods for correcting a severe flexion deformity of the knee during total knee arthroplasty can often lead to the excessive release of the posterior capsule and medial or lateral collateral ligament [28]. As many reports on flexion contracture management in the RA knee are available in the literature, the peroneal nerve palsy in TKA was concerned previously [2,3]. Preoperative severe flexion contracture was assumed as the risk factor for the development of the nerve palsy after TKA [29,30]. In TKA, complete intraoperative correction of severe flexion deformity is dangerous, which can cause complications such as the peroneal nerve palsy [31]. At present study, the surgical decompression of peroneal nerve was not performed and the transient peroneal nerve palsy had recovered after conservative therapy. Thus, the good result should be due to the appropriate soft tissue balancing other than a massive release at the time of arthroplasty.

The success of TKA in severe flexion deformity of RA patients depends on many factors, including the preoperative condition of the joint, surgical technique, and postoperative rehabilitation [32-34]. Splints are good supportive devices in flexion patients. The experience of Sarokhan et al. [10] has shown that the use of preoperative and postoperative serial casts aids greatly in the correction of severe flexion deformity of the knee. The use of dynamic extension splinting at night is beneficial to improve flexion contractures in this case studies. Physiotherapy is another important component of flexion patients [33]. In this study, splints are supportive devices in flexion patients until the some residual flexion contractures were totally corrected. Rand [35] reported that the most important complication affecting the results of total knee replacement in patients with RA is infection. Rates of infection have been reported to be approximately three times greater in patients with RA than in those with OA [36,37].

## Conclusions

TKA can be performed successfully in RA knees with severe flexion contracture. It is very important in TKA to maintain the joint stability in the condition of severe flexion contracture deformity of the RA knee.

## Competing interests

The authors declare that they have no competing interests.

## Authors’ contributions

DY participated in the design of the study and drafted the manuscript. FP participated in the design of the study and coordination and helped to draft the manuscript. JY participated in the design of the study and performed the statistical analysis. All authors read and approved the final manuscript.
